# Singular Olive Oils from a Recently Discovered Spanish North-Western Cultivar: An Exhaustive 3-Year Study of Their Chemical Composition and In-Vitro Antidiabetic Potential

**DOI:** 10.3390/antiox11071233

**Published:** 2022-06-23

**Authors:** María Figueiredo-González, Lucía Olmo-García, Patricia Reboredo-Rodríguez, Irene Serrano-García, Glenda Leuyacc-del Carpio, Beatriz Cancho-Grande, Alegría Carrasco-Pancorbo, Carmen González-Barreiro

**Affiliations:** 1 Food and Health Omics, Department of Analytical and Food Chemistry, Faculty of Science, University of Vigo-Ourense Campus, 32004 Ourense, Spain; mariafigueiredo@uvigo.es (M.F.-G.); glenda.ro.delcarpio@gmail.com (G.L.-d.C.); bcancho@uvigo.es (B.C.-G.); cargb@uvigo.es (C.G.-B.); 2 Department of Analytical Chemistry, Faculty of Science, University of Granada, Av. Fuentenueva s/n, 18071 Granada, Spain; iserrano@ugr.es (I.S.-G.); alegriac@ugr.es (A.C.-P.)

**Keywords:** autochthonous cultivar, virgin olive oil, phenolic compounds, diabetes mellitus, α-glucosidase inhibition, multivariant chemometric tools

## Abstract

In this work, the quality and physicochemical parameters, phenolic composition, and antidiabetic potential of olive oils obtained from olives belonging to centenarian olive trees of the so-called ‘Mansa de Figueiredo’ cultivar were evaluated during three consecutive crop seasons (2017–2019). The oils produced during the three crop years were classified as extra virgin based on the quality-related indices, sensory analysis, and the genuineness-related parameters. In addition, LC-ESI-TOF MS was used to get a comprehensive characterisation of the phenolic fraction while LC-ESI-IT MS was applied for quantitation purposes. The content of phenolic compounds (ranging from 1837 to 2434 mg/kg) was significantly affected by the harvest year due to the environmental conditions and ripening index. Furthermore, although significant differences in the inhibitory effects against the α-glucosidase enzyme for the EVOOs extracted throughout the three successive years were detected, all the studied EVOOs exhibited a stronger inhibitor effect than that found for acarbose.

## 1. Introduction

The existence of ancient olive trees in Galicia (NW Spain), since the arrival of Romans to the Iberian Peninsula, has favoured their adaptation to the edapho-climatic conditions that characterise this area, and their resistance to undergo several diseases. These ancient trees, considered a biodiversity reservoir of minor cultivars, represent a very important genetic heritage of Galicia that is at risk of being lost. For that reason, it is imperative to look for ways to exploit and valorise them.

In this sense, an emerging interest in the Spanish scientific community has been growing in the last few years. Several reports have been published, for example, (i) reporting the localisation of ancient olive trees in Galicia, their characterisation using botanical and molecular markers, and examining whether or not these trees represent unknown native genotypes [1]; (ii) implementing the scientific basis for the creation of a Protected Designation of Origin (PDO) [2]; (iii) evaluating the consumer acceptance of commercial EVOOs elaborated with autochthonous Galician cultivars [3]; and (iv) evaluating simple sequence repeats (SSR) and single-nucleotide polymorphism (SNP)-based methods in two autochthonous varieties, and their potential for integration in a microfluidic device [4].

Several years ago, the prospecting and collection of olive plant material from the main olive-growing area of Galicia, carried out by our research group, allowed us to identify an autochthonous olive cultivar (namely by the authors ‘Mansa de Figueiredo’) using an effective protocol for characterising, identifying, and authenticating olive plant material based on morphological and molecular (SSR markers) traits [5].

Although the total productivity of this cultivar is usually low (basically due to the limited number of catalogued trees), the production of some types of singular and unique olive oils from traditional varieties located in specific geographical areas could satisfy the demand for differentiated products in a rising competitive market, with the possibility to apply the EFSA Health Claim on them [6,7]. In fact, a study from our research group verified that the extra virgin olive oils (EVOOs) obtained from the autochthonous Galician variety ‘Mansa de Figueiredo’ stand out for their noteworthy sensory, nutritional, and health-promoting properties [8]. Moreover, these olive oils have shown a high content of phenolic compounds and they will presumably meet the criteria to hold the approved EFSA Health Claim on secoiridoid derivatives.

The many benefits of the phenolic compounds from the olive oils that counteract multiple diseases (including cancer, atherosclerosis, liver steatosis and/or other liver tissue damage, cardiovascular diseases, amyloid and neurological diseases, obesity, and type 2 diabetes mellitus (DM2), between others) are widely known [9].

The absorption of glucose in the small intestine occurs by the action of the α-glucosidase enzyme, leading to a fast increase in blood glucose levels [10,11,12]. In this sense, diet management might be a novel and comprehensive dietary approach for controlling DM2 by enzyme inhibition, with the advantage of reducing the side effects produced by the anti-diabetic therapeutic drugs currently available (acarbose, miglitol, voglibose) [13].

There are some characteristic phenolic compounds from olive oil that can act as α-glucosidase inhibitors [14,15,16,17,18]. As far as we are aware, very few previous works proved the inhibitory activity against digestive enzymes (α-glucosidase and α-amylase) of phenol-rich EVOO extracts from the Italian varieties ‘Frantoio’, ‘Ortice’, and ‘Ortolana’ [15] and the Spanish varieties ‘Cornicabra’ and ‘Picual’ [19]. Recently, the hypoglycaemic effect of autochthonous Galician EVOO (obtained on an industrial scale) was discovered. The phenolic compounds from these olive oils act as powerful inhibitors of α-glucosidase, stronger than the commercial inhibitor acarbose [12]. Moreover, it was also verified that the ripeness of the olives might not be a decisive factor in the antidiabetic potential of the olive oils from the autochthonous Galician variety ‘Brava Gallega’ [20].

The concentration and composition of phenolic compounds in olive oil are strongly affected by a wide range of factors. As intrinsic factors, the genotype stands out above all; the genetic origin of olives seems to be the main factor responsible for the high variation in their concentration [21,22]. Extrinsic factors, such as agronomical, environmental, edaphic, and technological, are the most relevant, with special emphasis on the ripening stage of olives at the harvesting time, geographical location of the cultivar [23], crop season [24], industrial processing techniques, storage conditions, and cultivation systems [20,23,25,26,27].

In this context, this work aims to characterise, for the first time, olive oils elaborated with the so-called Galician autochthonous variety ‘Mansa de Figueiredo’ throughout three consecutive crop years (2017, 2018, and 2019). Physicochemical parameters (viz. quality and purity parameters) and sensory attributes were evaluated to check whether they could be classified within the highest-quality commercial category, “Extra Virgin”. The profile of phenolic compounds of these olive oils was thoroughly characterised and the EFSA requirement related to the content of phenolic compounds was also verified. Additionally, an evaluation of the antidiabetic capacity of ‘Mansa de Figueiredo’ olive oils throughout the three successive seasons was carried out.

## 2. Materials and Methods

### 2.1. Chemicals and Reagents

Folin-Ciocalteu (FC) reagent, sodium carbonate (Na_2_CO_3_), water HPLC grade (H_2_O), gallic acid (GA), methanol (MeOH), Trolox, 2,2-diphenyl-1-picrylhidrazyl (DPPH), ethanol (EtOH), and sodium molybdate dihydrate (Na_2_MoO_4_·2H_2_O), employed for spectrophotometric analysis, were supplied by Sigma-Aldrich (St. Louis, MO, USA).

α-Glucosidase (=maltase from Saccharomyces cerevisiae), 4-nitrophenyl α-D-glucopyranoside (PNP-G), potassium dihydrogen phosphate (KH_2_PO_4_), and sodium hydroxide (NaOH) were obtained from Sigma-Aldrich (St. Louis, MO, USA).

Acetonitrile (ACN) and MeOH LC-MS grade were both acquired from Prolabo (Paris, France). Deionized water was obtained by using a Milli-Q system from Millipore (Bedford, MA, USA). Acetic acid (AcH) for acidification of aqueous mobile phase and commercially available pure standards (apigenin (Api), ferulic acid (Fer), hydroxytyrosol (HTy), luteolin (Lut), oleuropein (Ol), pinoresinol (Pin), *p*-coumaric acid (*p*-Cou), quinic acid (Quin), tyrosol (Ty), and vanillic acid (Van)) were purchased from Sigma-Aldrich. 3,4-dihydroxyphenylacetic acid (DOPAC), used as an internal standard (IS) for the phenolic profile determinations, was acquired from Sigma-Aldrich. Stock solutions for each analyte were prepared by dissolving the appropriate amount of each chemical standard in ACN:H_2_O (50:50, *v*/*v*). Following this, they were serially diluted to prepare the working solutions covering concentration levels within a range 0.1–50 mg/L. All the samples and stock solutions were filtered through a ClarinertTM 0.22 μm nylon syringe filter from Agela Technologies (Wilmington, DE, USA) and stored at −20 °C until further analysis.

### 2.2. Sampling

The olive trees studied were grown in three nearby traditional olive groves located in the northwest of Spain (Galicia), and managed with the same agronomic techniques, under non-irrigated organic agricultural practices (Orchard 1: N 42°23′20.2308″, W 7°12′14.6736″; Orchard 2: N 42°23′17.502″, W 7°12′18.162″; Orchard 3: N 42°24′25.6968″, W 7°13′38.5824″) (Figure S1, Supplementary Materials).

These groves include old trees from diverse cultivars, ʻMansa de Figueiredoʼ being one of them. The olive trees from ‘Mansa de Figueiredo’ cultivar were carefully selected taking into account the tree appearance, structure, and trunk thickness, which are classical indicators of the tree age, and the included information in a previous study conducted to achieve their genotypic and phenotypic identification [5].

The same selected trees were harvested over three consecutive crop seasons (2017, 2018, and 2019) to minimise the effect of the edaphic factors. Olive fruit sampling was carried out according to the International Olive Council (IOC) recommendations in the month of November of each year (8 November 2017, 27 November 2018, and 27 November 2019). The ripening index (RI) was determined as described by Estación de Olivicultura of Jaén, Spain [28].

A monthly record of the most determinant climatic parameters for the studied area over the selected period of three years (i.e., 2017–2019) is depicted in Figure S2 (Supplementary Materials). The data were collected from the closest governmental meteorological station to this area located in San Clodio (Ribas de Sil, Lugo, NW Spain) [29]. As seen in Figure S2, the 2017 climate year greatly differed from the other two; important differences in total precipitations and average temperatures (minimum and maximum) per month were observed, especially in the months of olive fruit growing and before olive harvesting.

### 2.3. Olive Oil Extraction

Approximately 20 kg of olive fruit was carefully hand-picked from the selected trees each harvesting season. After every sampling, healthy olive fruits were randomly selected and inspected to detect damaged fruits attacked by pests or infected by diseases (all the olives were found to be in undamaged state).

The fruits were processed in the first 24 h after harvest and monovarietal oils were extracted in triplicate (genuine replicates) using a pilot extraction plant consisting of an Abencor analyser (MC2 Ingeniería y Sistemas, Seville, Spain) equipped with a centrifugal machine, a hammer mill and a thermo-mixer, that reproduces the industrial process of VOO production at a laboratory scale. The olive milling was carried out at 3000 rpm with a 5 mm sieve. The olive paste malaxation was accomplished at 26 °C over 40 min with the addition of warm water (10%). The oil was separated in a basket centrifuge at 3500 rpm for 90 s. After centrifugation, the oil was decanted, filtered, and finally stored in dark glass bottles in the dark at −20 °C, without headspace, until further analysis.

### 2.4. Olive Oil Characterisation

#### 2.4.1. Quality Parameters

Free acidity, peroxide value, and specific UV spectrophotometric indices (K_232_ and K_270_) were evaluated by the analytical methods described in the Commission Regulation (EEC) No 2568/91 and its subsequent amendments [30]. K_232_ and K_270_ extinction coefficients were measured with an UV-Vis spectrophotometer (Eppendorf, Hamburg, Germany).

The sensory analysis of olive oils was carried out by ten expert tasters according to the official method of the IOC (IOC/T.20/Doc. No 15/Rev. 10 2018) [31] within the framework of EU Regulations (1348/2013, 2015/1833, 2016/1227, 2019/1604) [32,33,34,35]. The tasters evaluated positive gustatory (bitter), olfactory–gustatory (fruity), and tactile (pungent) attributes, as well as negative attributes (acid-sour, fusty/muddy sediment, frostbitten olives, musty-humid-earthy, rancid, winey-vinegary, among others). Besides, they were requested to assign positive olfactory descriptors within those listed in IOC/T.20/Doc. N° 22 November 2005 [36].

The oxidative stability (OS) of each EVOO was evaluated under accelerated oxidation conditions using the Rancimat apparatus following the protocol of Mancebo-Campos et al. [37].

#### 2.4.2. Fatty Acid, Sterol, and Triterpene Dialcohol Composition

The chemical composition was evaluated applying the analytical methods collected in the Commission Regulation (EEC) 2568/91 [30] and its subsequent amendments, establishing authenticity criteria for EVOOs.

Fatty acids, sterols, and triterpene dialcohol composition were analysed by gas chromatography-flame ionization detection (GC-FID) (Thermo Fisher Scientific, Waltham, MA, USA).

#### 2.4.3. Tocopherol Composition

α-, β-, γ-, and δ-tocopherol were determined following the IUPAC 2.432 method [38]. The found concentrations were expressed as mg of each tocopherol/kg oil.

Tocopherol compositions were determined by high-performance liquid chromatography-fluorescence detection (HPLC-FLD, Agilent Technologies, Waldbronn, Germany).

#### 2.4.4. Total Phenols, o-Diphenols, and Antioxidant Capacity

Olive phenolic compounds were extracted with MeOH:H_2_O (80:20, *v*/*v*) following the IOC method (IOC/T.20/Doc No 29) [39]. The total phenolic content and *o*-diphenols content of the extracts were evaluated according to the spectrophotometric methods described by Reboredo-Rodríguez et al. [8], using a GA calibration curve (R^2^ = 0.999). Both contents were expressed as mg of GA/kg oil.

The antioxidant capacity was determined by using the 2,2-diphenyl-1-picrylhydrazyl (DPPH•) radical scavenging method, according to Gorinstein et al. [40], with some modifications. The results are expressed as μmol Trolox equivalents/kg oil.

### 2.5. Evaluation of the Phenolic Profile

#### 2.5.1. Extraction Protocol

Phenolic compounds were isolated from the olive oil samples by using a liquid–liquid extraction protocol previously reported by Bajoub et al. [41], with some modifications. Briefly, 2 (±0.01) g of EVOO was weighed in a conical centrifuge tube (15 mL) and spiked with 25 µL of the IS from a methanolic stock solution at a concentration of 500 mg/L. The sample was dissolved with 1 mL of *n*-hexane, after solvent evaporation under N_2_ current, and was extracted three times with 2 mL portions of the mixture MeOH:H_2_O (60:40, *v*/*v*) by vigorous vortex shaking. All of the supernatants obtained after centrifugation were combined and evaporated to dryness with a TurboVap Evaporator. Lastly, the remaining residue was redissolved in 1 mL of ACN:H_2_O (50:50, *v*/*v*). Before injection into the chromatographic system, an aliquot of the prepared extract was diluted (1:10, *v*/*v*) with ACN:H_2_O (50:50, *v*/*v*) and filtered through a 0.22 µm nylon syringe filter.

#### 2.5.2. LC-MS Analysis

The LC-MS analyses were performed on an Agilent 1260 LC system (Agilent Technologies). It was coupled to a Bruker Daltonics Esquire 2000™ ion trap mass spectrometer (Bruker Daltonik, Bremen, Germany) by means of an electrospray ionization source.

The separation was carried out in a Zorbax C_18_ analytical column (4.6 × 150 mm, 1.8 μm particle size) (Agilent Technologies) operating at 25 °C, according to the method proposed by Bajoub et al. [41]. Analytes of interest were eluted with a mobile phase gradient of acidified water (0.5% AcH, phase A) and ACN (phase B) at a flow rate of 0.8 mL/min. Regarding the MS conditions, the ion trap was operated in full-scan mode (*m*/*z* range 50–800) in negative polarity. Moreover, MS/MS analyses were also performed to characterise the fragmentation patterns of the studied compounds.

Chromatographic data acquisition was carried out by using ChemStation B.04.03 software (Agilent Technologies). The mass spectrometer was controlled by using the software Esquire Control. The obtained files were processed with the software Data Analysis 4.0 (Bruker Daltonik).

The identification of the phenolic compounds found in the olive oil samples was based on: (i) the use of pure standards (when commercially available); (ii) relative retention times; and (iii) the comparison of the MS and MS/MS spectra with previously published results. Calibration curves for each pure standard were built using different concentrations of the standard mixture solution and plotting peak areas vs. concentration levels. When a pure standard was not available, the quantification was made using the calibration curve of a similar (or structurally related) compound: (i) HTy was used for oleuropein aglycon (OlAgl) and related compounds; (ii) Ty was used for ligstroside aglycon (LigAgl) and related compounds; (iii) Lut was used for diosmetin (Dios); and finally, (iv) oleuropein was used for all elenolic acid (EA) derivatives.

### 2.6. Assessment of the In-Vitro Antidiabetic Activity

The extracts obtained following the conditions specified in Section 2.5 were evaporated and re-dissolved in phosphate buffer as a previous step to be subsequently used in the in-vitro inhibitory assay. α-Glucosidase inhibitory activity was assessed by following a reported procedure [11]. Briefly, each reservoir was filled with PNP-G (2.5 mM), phosphate buffer, and extract (or buffer in the case of negative control). The reaction was initiated by adding an enzyme solution (0.28 U/mL). The plates were incubated at 37 °C for 10 min. The rate of release of 4-nitrophenol from PNP-G at 405 nm was measured in an LT-5000 MS Elisa Reader (Labtech International Ltd., East Sussex, UK) from 0 to 10 min. Acarbose was established as the positive control. The concentration of the olive oil extracts varied from 31 to 1000 µg of dry extract/mL.

### 2.7. Statistical Analysis

Comparisons between means were performed by applying One-Way Analysis of Variance (ANOVA) with Tukey’s post-hoc test, after verifying that data normality and homoscedasticity conditions were fulfilled using Shapiro–Wilk and Levene tests, respectively. The differences between crop years were considered significant with *p* < 0.05.

Additionally, a set of multivariate data analysis (viz. principal component analysis (PCA), hierarchical clustering analysis (HCA), and linear discriminant analysis (LDA)) were performed on phenolic compound quantitative data to assess the potential of these substances to discriminate the studied samples according to the crop season.

All statistical tests were carried out with the software package Statgraphics Centurion XVI from StatPoint Technologies Inc (Suite, VA, USA).

## 3. Results

For the first time, an exhaustive study of the inter-annual variability in the content of the most relevant compounds related to the quality and genuineness of ‘Mansa de Figueiredo’ olive oils was carried out.

At this point, it is important to note that the variety reported herein is genetically different from ‘Mansa Gallega’, which is one of the two Galician varieties currently recognized by the Spanish Department of Agriculture [42]. As stated before, extensive efforts are being put into the identification of other varieties [1,2]. Up to now, 20 varieties have been discovered and the process of protection following the UPOV (International Union for the Protection of New Varieties of Plants) system has already been initiated for some of them—i.e., ’Brava Gallega’, ’Mansa Gallega’, ’Brétema’, ’Carapucho’, ’Carmeliña’, ’Folgueira’, ’Hedreira’, ’Maruxiña’, ’Santiagueira’, ’Susiña’, and ’Xoana’ [43].

When this study was designed, the peculiarities of ‘Mansa de Figueiredo’ cultivar (growing practices) had to be taken into account. ‘Mansa de Figueiredo’ olive trees were spread in a reduced steep area of traditional olive groves cultivated on terraces, with low numbers of ancient trees per hectare and very limited production. The month of November is consistently selected for harvesting by olive growers in this region, since delaying the harvest period to December would mean dealing with frost and snowfalls, which would be detrimental to the quality of olives and, consequently, of the oil. In this regard, the factor “crop season” encompasses not only the climatic differences but also the yearly variation in the ripeness: RI (2017) = 3.5 ± 0.2, RI (2018) = 5.1 ± 0.1, and RI (2019) = 2.3 ± 0.2.

Therefore, the main objective of this study was to evaluate if the influence of extremely different (climatologically) crop seasons could significantly diminish the quality of the oils, with special emphasis on their functional quality. In other words, the oils were intended to be as representative as possible of the actual production of the olive mills of the evaluated area over the three studied seasons.

Thus, olive oils generated from *Olea europea* L. var. ‘Mansa de Figueiredo’ (MF-17, MF-18, and MF-19) were chemically examined, as described below. Table 1 summarizes the results obtained for all the evaluated quality parameters, sensory attributes, the profiles of fatty acids, sterols, triterpenic alcohols, and tocopherols, as well as the limits for the regulated quality standards.

### 3.1. Effect of Crop Year on Quality-Related Parameters and Sensory Attributes

**Quality-related indices.** The free acidity showed no statistically significant differences for the olive oils obtained in different harvesting seasons and the peroxide value, indicative of oxidation, was around 3.2 mequiv O_2_/kg. Both parameters were within the limits set by the EU for Extra Virgin Olive Oils at the three crop years (Commission Implementing Regulation (EU) 2019/1604 [35]).

Regarding spectrophotometric UV absorption values at 232 nm (K_232_) and 270 nm (K_270_), the K_232_ index was statistically different in the 2017 harvest; meanwhile, the K_270_ index remained statistically invariable throughout the three years. Both K_232_ and K_270_ indices were below the limits 2.50 and 0.22, respectively, established for the Extra Virgin category.

Despite the significant differences found in some of the just-mentioned parameters, the quality-related indices levels were generally low, indicative of scarce oxidation of the oils. This fact was logically expected, keeping in mind that the olive oils were obtained from hand-picked fresh fruit and extracted in a short time after harvesting, minimizing the risk of hydrolysis and oxidation of fatty acids.

**Sensory attributes.** Sensory analysis is an important tool to classify olive oils in different commercial categories. The results of the sensory analysis of ‘Mansa de Figueiredo’ olive oils of the three successive crop years are shown in Table 1.

The fruity notes ranged from 4.0 to 4.6, bitter attributes were found among 3.4 and 4.0, and pungency varied between 4.1 and 4.5. In addition, the median of defects was equal to 0.0. Therefore, the analysed olive oils can be classified as “Extra Virgin Olive Oil”. According to the intensity of perception, as specified by EU legislation (Commission Implementing Regulation, 2019/1604 [35]), the positive attributes were “medium” (i.e., the median of attributes is more than 3.0 and less or equal to 6.0) and their scores were ‘‘well balanced” (i.e., the median of the pungent attribute is not more than 2.0 points above the median of the fruitiness).

In a subsequent additional descriptive taste analysis, the panellists found common attributes throughout seasons, namely: “grass”, “almond”, and “olive leaf”. The “Artichoke” descriptor was exclusive for the 2017 olive oils; meanwhile, “apple” and “dried fruit” stood out in the ripest 2018 fruity oils. Finally, the “tomato” descriptor was only appreciated in the MF-19 olive oil.

### 3.2. Effect of Crop Year on Olive Oil Genuineness-Related Parameters

**Fatty acid composition.** The fatty acid profile provides information about genuineness of olive oils and is related to its health-promoting properties [44,45].

As expected, the main fatty acids detected in all olive oils samples, independently of the crop year, were (in decreasing order of abundance): oleic acid (C18:1), palmitic acid (C16:0), linoleic acid (C18:2), and stearic acid (C18:0) (Table 1). They accounted for approximately 97% of the total fatty acid composition. These major compounds, together with palmitoleic acid (C16:1), arachidic acid (C20:0), and behenic acid (C22:0), differed significantly among crop years.

In any case, all percentages of fatty acids fell within the recommended ranges for EVOO set by EU Regulation (Commission Implementing Regulation, 2019/1604 [35]).

As previously detailed, the profile of fatty acids varied according to harvest season. Among environmental factors, low temperatures are supposed to lead to an increase in the content of unsaturated fatty acids, such as linoleic and linolenic acid contents. However, in our case, no relationship between temperature and unsaturated fatty acids could be established. Besides temperature, other factors, such as latitude, climate, fruit ripening stage, storage, and processing conditions, could also affect the fatty acid content [44,46].

The fatty acid composition of olive oils elaborated from the ‘Mansa de Figueiredo’ variety for three successive crop years was characterised by a high monounsaturated content (ranging from 75.74% to 79.55%) and a notable proportion of polyunsaturated fatty acids (6.68%–9.46%). The ratios ∑MUFA/∑PUFA and C18:1/C18:2 (8.00–11.92 and 8.40–13.05, respectively) can help to catalogue the oils by the cultivar classification proposed by Zarrouk et al. [47]. In this sense, ‘Mansa de Figueiredo’ olive oil fell within the group with high ∑MUFA/∑PUFA (5.9–17.5) and C18:1/C18:2 ratios (6.3–21.5), which includes the following cultivars: ‘Cayon’, ‘Changlot Real’, ‘Coratina’, ‘Cornezuelo’, ‘Koroneiki’, ‘Leccino’, ‘Lechín de Granada’, ‘Olivière’, and ‘Verdial de Vélez-Málaga’ [5].

**Sterol and triterpene dialcohol composition.** The sterol content (represented by cholesterol, brassicasterol, campesterol, stigmasterol, apparent β-sitosterol, and Δ7-stigmastenol) in the monovarietal ‘Mansa de Figueiredo’ olive oils under study is also shown in Table 1.

The main compound belonging to this family was apparent β-sitosterol, comprising approximately 95% of total sterols detected. Having a look at Table 1, a tendency for the sterol levels (individually or as a sum) to decline with an increased ripeness index is observed.

The triterpenic dialcohols (erythrodiol and uvaol) are concomitantly analysed with the sterol fraction because they are also a part of the unsaponifiable fraction. Similarly, it a downward trend was generally noted for these compounds when the ripeness index increased. These findings agree with the results of Boulkroune et al. [48].

### 3.3. Effect of Crop Year on Other Quality-Related Parameters Not Included in Current European Regulations

Among the natural antioxidants present in virgin olive oil, tocopherols stand out because of their antioxidant activity and nutritional value.

Four different tocopherols have been described as predominant in virgin olive oil: α-tocopherol, β-tocopherol, γ-tocopherol, and δ-tocopherol. α-Tocopherol was the most abundant compound for the selected olive oils, ranging from 184 to 303 mg/kg. The levels of α-tocopherol as well as the total tocopherols content were higher for the EVOO elaborated in 2018 and 2019 than those for the 2017 crop season. It has been previously observed that tocopherols (mainly α-tocopherol) generally decrease during the ripening process, and this is related to chlorophyll depletion, although the degree of reduction is logically cultivar specific [49]. On the other hand, tocopherol composition is heavily influenced by harvest year [44]. In fact, it is well known that in addition to the genetic factor, tocopherol content can be influenced by climatic parameters, mainly temperature, precipitations, and altitude [50]. In our case, it seems plausible that the cause of the observed fluctuations in the data could be the great difference in the climatic conditions for the year 2017 with respect to the rest (Figure S2, Supplementary Materials).

### 3.4. Effect of Crop Year on Olive Oil Phenolic Compounds

Table 2 shows the phenolic composition of olive oils obtained from the ‘Mansa de Figueiredo’ variety for three consecutive crop years (assessed using both spectrophotometric and chromatographic methods).

#### 3.4.1. Total Phenolic Content by Spectrophotometric Methods

**Total phenolic content.** Folin-Ciocalteu (FC) spectrophotometric assay is, without any doubt, the most widely employed and widespread method to determine the total phenolic content in food matrices, but, as a most remarkable drawback, it cannot give information on the chemical nature of the different compounds belonging to the phenolic fraction.

As can be seen in Table 2, the total phenolic content for ‘Mansa de Figueiredo’ EVOOs varied from 675 to 793 mg GA/kg oil and the differences are only statistically significant for the 2019 crop year, with the highest total concentration. According to the classification established by Servili et al. [27], the tested EVOOs herein can be considered as high-phenolic olive oils, regardless of both the crop year and the RI (in the classification referred to, the oils are categorized as high-phenolic-content VOOs > 500 mg GA/kg oil; medium content VOOs from 250 to 500 mg GA/kg oil; and low content VOOs < 250 mg GA/kg oil). The variation in phenolic compounds considering several seasons is in accordance with other works described in the literature [44,51,52,53,54]. Climatic conditions (temperature and rainfall), but, most importantly, olive fruit RI, could be responsible for the significant differences in the total phenolic content observed in the selected olive oils [20,44,55].

These values are in the same order of magnitude as those reported for EVOOs obtained from the Cornicabra variety (633 mg GA/kg oil [56]; 556 mg GA/kg oil [25]; and 680 mg GA/kg oil [24]), and Picual variety (605 mg GA/kg oil [25]), both considered as the Spanish cultivars with the highest total phenolic content [57]. In addition, Nevadillo y Villalonga Spanish varieties (609 and 700 mg GA/kg oil, respectively), recently introduced in Argentina [58], and the Koroneiki variety, grown in Southern Spain (994 mg GA/kg oil [59]), also exhibited similar total phenolic content.

Other recognized Spanish varieties widely cultivated, such as Manzanilla (234 mg GA/kg oil), Picudo (207 mg GA/kg oil), Hojiblanca (169 mg GA/kg oil), and Arbequina (153 mg GA/kg oil) [57], stand out for having a lower phenolic content than Mansa de Figueiredo EVOOs.

***o*-Diphenol content.** *o*-Diphenol content in the oil (HTy and secoiridoid derivatives of oleuropein aglycone) is always much lower than that of total phenolic compounds. However, the bioactivity of this subgroup of polyphenols may even be higher due to the *ortho*-position of the -OH groups. The *o*-diphenol content of the selected olive oils (197–226 mg GA/kg oil) remained practically invariable through the three successive crop years, regardless of the RI of the harvested olives. Data obtained herein was very similar to the values obtained for Picual (206 mg GA/kg oil [60], and 243 mg coumaric acid/kg oil [56]) and Manzanilla (219 and 316 mg coumaric acid/kg oil olive oils [56].

**Antioxidant capacity.** The antioxidant capacity was assessed by determining the hydrophilic phenolic compounds’ ability to scavenge free DPPH radicals (Table 2). High antioxidant capacities for the selected olive oils were registered (1963 to 2678 µmol Trolox/kg oil). Only MF-17 olive oil (with RI = 3.5 and the lowest total phenolic content) was statistically different when compared to the other EVOOs. A possible explanation for the inverse relationship between total phenolic content and antioxidant capacity in MF-17 olive oil could be due to the presence of other non-phenolic compounds and the different contribution of the individual phenolics to the antioxidant capacity [20].

Borges and co-workers [61] pointed out that the climatic conditions could affect the antioxidant potential of oils, but differently depending on cultivars. The antioxidant capacities evaluated herewith were higher than those of Picual olive oils (236–794 µmol Trolox/kg oil [62], but similar to those described for Cornicabra olive oils (1877 µmol Trolox/kg oil [19]).

In summary, the tested olive oils showed high antioxidant capacity through several crop years, regardless of the olive fruits RI.

#### 3.4.2. Phenolic Content by LC/MS-MS

A validated LC/MS-MS profiling method, developed by Bajoub et al. [41], allowed the separation and identification of the main phenolic compounds from the studied ‘Mansa de Figueiredo’ EVOOs.

Figure S3 (Supplementary Materials) shows a typical LC-ESI-IT MS profile of a phenolic extract of ‘Mansa de Figueiredo’, where the extract ion chromatograms (EICs) of the determined compounds are depicted. Up to 21 phenolic compounds, as well as quinic acid and EA derivatives (a series of polar non-phenolic compounds), were detected in ‘Mansa de Figueiredo’ olive oils. All the identified compounds are presented in Table 2 together with their deprotonated molecular ion [M − H]^−^, retention time, and concentration levels (expressed as mg/kg olive oil ± standard deviation). The phenolic compounds were classified according to their chemical structure and they were grouped into several families, i.e., secoiridoids (oleuropein and ligstroside derivatives), simple phenols, flavonoids, organic acids, and lignans. EA derivatives and quinic acid, which are non-phenolic but structurally related compounds, were grouped together. Although the phenolic profile of the studied olive oils did not vary qualitatively among the three harvest years, marked differences in the concentration of several compounds were observed. The subsequent paragraphs include a comprehensive description of the results found for each chemical class.

**Secoiridoid derivatives.** This group is constituted by aglycon forms of the secoiridoid glucosides formed during oil extraction by β-glucosidase enzymatic hydrolysis of oleuropein, demethyloleuropein, and ligstroside [63]. Secoiridoids were the main phenolic group in the studied olive oils, which is in agreement with the findings of other authors regarding various monovarietal olive oils [19,64]. The highest secoiridoids concentration value was observed for MF-19 olive oil, obtained during the 2019 harvest season (1938 mg/kg oil), whereas the lowest concentration was found for samples from the 2018 crop season (694 mg/kg oil). This group is divided into two sub-categories: oleuropein derivatives, including decarboxymethyl oleuropein aglycone (DOA, also known as oleacein), hydroxy decarboxymethyl oleuropein aglycone (HDOA), dehydro oleuropein aglycone (DH-OlAgly), and three oleuropein aglycone isomers (OlAgl (Is I), OlAgl (Is II), and OlAgl (Is III)); and ligstroside derivatives, including decarboxymethyl ligstroside aglycone (DLA, also known as oleocanthal) and three ligstroside aglycone isomers (LigAgl (Is I), LigAgl (Is II), and LigAgl (Is III)).

The total concentration of oleuropein derivatives (quantified in terms of HTy), ranged from 354 to 603 mg HTy/kg oil, whilst ligstroside derivatives (quantified as Ty) varied between 340 and 1334 mg Ty/kg oil.

Regarding oleuropein derivatives, the most abundant was DOA, with a mean concentration of 350, 221, and 542 mg HTy/kg oil for MF-17, MF-18, and MF-19 EVOOs, respectively. The decrease in DOA in MF-18 compared to the rest of EVOOs could be ascribed to the esterase activity responsible for oleuropein degradation as ripening time advanced [65].

Other oleuropein derivatives also found at relatively high concentration levels were the three oleuropein aglycone isomers (specifically, the main peak with amounts ranging from 36 to 93 mg HTy/kg oil).

Considering ligstroside derivatives, DLA showed the highest concentrations in the studied oils: 704, 120, 1061 mg Ty/kg oil for MF-17, MF-18, and MF-19 EVOOs, respectively. The lowest concentration of DLA in MF-18 compared to the rest of EVOOs could be explained in the same way as for DOA. This abatement in oleocanthal levels along the ripening process in VOOs was also observed by Goméz-Rico et al. [66]. This compound seems to be the most strongly affected by the harvest year. These results are in good agreement with a previous work where the concentration of DLA examined in EVOOs of 25 different varieties, elaborated by means of an Abencor system, was greatly dependent on crop season [22]. The three isomers of ligstroside aglycone—relevant compounds of the phenolic profile of any VOO—were found at lower concentration levels in comparison to those of DLA (the main peak exhibited a mean content of 200 mg Ty/kg oil for all seasons).

Within the group of the non-phenolic compounds, EA was the most abundant compound, and it underwent important changes from year to year (206, 123, and 431 mg oleuropein/kg oil for MF-17, MF-18, and MF-19 EVOOs, respectively). In addition, quinic acid was found within a range of 0.17–0.40 mg/kg oil in the analysed samples.

**Simple phenols.** Oxidized hydroxytyrosol (O-HTy), HTy, hydroxytyrosol acetate (HTy-Ac), and Ty represented the second-highest content group of phenolic compounds (comprising 22% of total phenolics) in the tested EVOOs. A plausible reason explaining the content observed for this group could be the freshness of the studied olive oils (a short time passed since their elaboration). It is widely known that their concentrations increase over time through the hydrolysis of secoiridoids [67]. The tested samples showed average concentrations for total simple phenols of 38, 67, and 10 mg/kg for MF-17, MF-18, and MF-19 EVOOs, respectively. The concentration of HTy (18, 19, and 3 mg HTy/kg oil) and HTy-Ac (10, 27, and 5, quantified in terms of mg HTy/kg oil) were higher than Ty (10, 6, and 2 mg Ty/kg oil) for the EVOOs extracted in the three successive crop seasons. It is worth noting that both simple phenols, HTy and HTy-Ac, are widely known for their biological activity [68,69]. The observed high concentration of total simple phenols in MF-18 compared to the rest of the EVOOs could be explained, in part, by secoiridoid degradation during fruit ripening [65].

The health claim approved by the European Food Safety Agency (EFSA) establishes that olive oil phenols contribute to the protection of blood lipids from oxidative stress for olive oils containing at least 5 mg of HTy and its derivatives (e.g., oleuropein complex and Tyr) per 20 g of olive oil [70]. The contents of HTy and its derivatives per 20 g of olive oil, calculated as the sum of the individual phenols considering simple phenols and secoiridoids categories (32, 15, and 39 mg/20 g of olive oil for MF-17, MF-18, and MF-19, respectively) far exceed the EFSA threshold in all the evaluated seasons. The spectrophotometric method gave lower values (10, 10, and 12 mg/20 g of olive oil for MF-17, MF-18, and MF-19, respectively) but it is well known that the spectrophotometric methods underestimate the phenolic content if compared to the chromatographic methods [71]. In any case, the studied ‘Mansa de Figueiredo’ EVOOs presented a high phenolic content, fulfilling the EFSA requirements, regardless of the climatic conditions and the olive fruit RI in the three evaluated crop years. Thus, the studied EVOOs could exhibit the specific health claim on the oil label.

**Flavonoids.** Lut, Api, and Dios constitute the third chemical group in terms of concentration levels. Lut was the most abundant flavone (mean concentration of 3 mg Lut/kg oil). The rest of the flavonoids were detected at lower concentrations (mean concentration of 0.5 mg Api/kg oil for Api and 0.3 mg Lut/kg oil for Dios). The average values of the total content of flavonoids were 1, 6, and 2 mg/kg oil for MF-17, MF-18, and MF-19 EVOOs, respectively. Again, MF-18 exhibited the highest flavonoid concentration, possibly due to the breakdown of their glucoside forms by glycosidase activity during olive ripening [65].

**Phenolic acids.** In the studied EVOOs, a hydroxybenzoic acid (Van) and two hydroxycinnamic acids (*p*-Cou and Fer) belonging to this chemical group were found. They were determined at relatively low levels (the total quantities were 0.04, 0.43, and 0.13 mg/kg from 2017 to 2019). These compounds have been associated with the colour and sensory qualities of foods and they have also been used as potential markers of geographical origin and olive cultivars [72].

**Lignans.** Pin was the only lignan quantified in the analysed samples with a mean concentration of 0.25, 0.47, and 0.16 mg Pin/kg oil for MF-17, MF-18, and MF-19 EVOOs, respectively.

As can be seen in Table 2, the total phenolic content for ‘Mansa de Figueiredo’ EVOO in the 2019 crop year (2434 mg/kg oil) was higher than that observed for the 2018 crop season (1008 mg/kg oil). An intermediate concentration was registered (1837 mg/kg oil) for MF-17 olive oil. As already indicated, the total phenolic content, in this case, was calculated by adding the individual concentration levels of each compound (each one quantified with respect to its pure standard—if available—or with respect to the most suitable standard among those available). This means that the total values are relative sums and serve mainly to compare samples. The qualitative and quantitative variability in phenolic compounds in EVOOs in our study could be mainly due to two factors: environmental conditions [26,44] and the different RI of olive fruit at harvesting time. Although in a general way, the EVOOs produced from green olives have higher phenolic content than those obtained from ripe fruits, the individual compounds can have different behaviour [20]. Therefore, in our case, it is evident that the crop season has a statistically significant effect on the studied EVOOs. These results are supported by previous works [52,53,61,64,73].

The comparison of the content of the phenolic compounds from ‘Mansa de Figueiredo’ EVOOs with others described in the bibliography is quite difficult and some considerations have to be taken into account. Firstly, the olive oil phenolic fraction can be determined with different analytical methods, including several detection techniques and quantification strategies (mostly using non-specific standards due to their lack of commercial availability) that lead to non-comparable results [71]. Secondly, the used oil extraction system can exert several changes on the olive oil phenolic fingerprint. In large-scale olive oil production, higher water volume is normally added to enhance the separation of oil from olive paste, and consequently, the hydrophilic phenolic compounds can be lost. In contrast, the addition of water in the lab-scale Abencor system is lower, preserving the phenolic fraction in the oil phase [22]. In fact, olive oils obtained at a large scale from the ‘Mansa de Figueiredo’ variety showed lower total phenolic content (882 mg/kg, determined by the same LC-MS method) compared to those obtained in this work [19].

Data herein are within the range for those obtained by Miho et al. [22], who evaluated the phenolic composition of EVOOs obtained from 80 cultivars (using the Abencor System), selected for their impact on worldwide olive oil production. However, if our results are compared, just for illustrative purposes, with other VOOs elaborated from Mediterranean varieties, such as Hojiblanca (366–473 mg/kg oil), Manzanilla (537 mg/kg oil), and Arbequina (178–388 mg/kg oil) [74], Cornicabra (633 mg/kg oil) and Manzanilla Cacereña (469 mg/kg oil) [25], Frantoio (336 mg/kg oil), Gentile (583 mg/kg oil), and Moraiolo (954 mg/kg oil) [75], a lower phenolic total content in all of them is observed.

#### 3.4.3. Chemometric Evaluation

With the purpose of seeking a possible discrimination among crop years based on the phenolic composition of the studied olive oils, several chemometric tools, including unsupervised methods (PCA and HCA) and supervised ones (LDA), were applied. Unsupervised methods give an overview of the dataset and try to identify general trends—without any prior knowledge—by grouping samples that show certain similarities. On the contrary, when supervised methods are applied, groups are known a priori and are used to build classification models that, at a later stage, will allow the allocation of new and unknown samples to the most probable class. The latter is very useful to point out markers that are significantly different between sample groups [76,77].

A first attempt was carried out by applying PCA to the standardized matrix data, which was constructed initially with 24 measured variables (the number of phenolic compounds that were quantified in the VOO samples) and 18 samples (3 olive oils × 3 crop years × 2 extraction replicates). Nevertheless, taking into account that the variables used to construct the principal components (PCs) must be correlated, non-useful variables from the matrix were eliminated after evaluating the Pearson correlation coefficients matrix and verifying if the correlation was statistically significant. In this sense, the number of variables was drastically reduced to 12 (grey shaded cells in Table 2).

**Figure 1:**
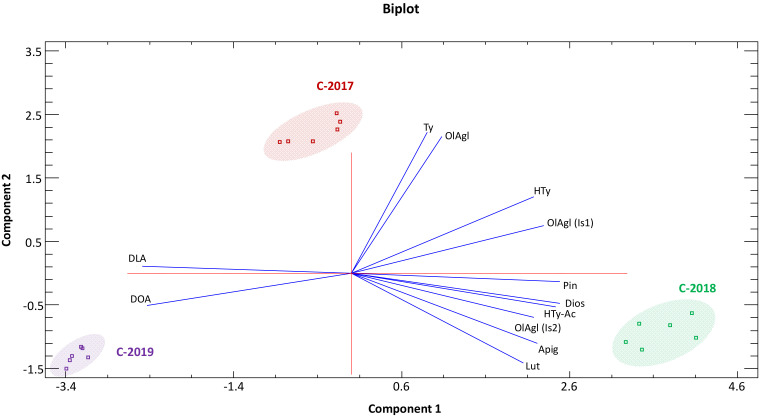
Distribution of ‘Mansa de Figueiredo’ EVOOs in a biplot system defined by the first two principal components (Component 1 vs. Component 2). C-2017: EVOOs from 2017 campaign; C-2018: EVOOs from 2018 campaign; C-2019: EVOOs from 2019 campaign. Loadings are shown as vectors.

The first two PC functions explained 94.86% of the data variability (PC1 and PC2 accounted for 72.46% and 22.39%, respectively). In Figure 1, a biplot (the superposition of score and loading plots) of PC1 vs. PC2 is shown. Such a figure shows how the EVOO samples are grouped based on their similarities and how the finally selected phenolic compounds influence this pattern. After having a look at this figure, it can be observed that the samples are clearly separated according to the crop season and each group is characterised by the concentration levels of several potential markers. In this sense, Ty and OlAgl were the phenolic compounds that mostly contributed to discriminate the EVOOs elaborated in the 2017 season. Two structurally similar secoiridoids, DOA and DLA, were those that most influenced the olive oil phenolic composition in 2019, while the remaining eight phenolic compounds were key to the complete discrimination achieved for the samples from 2018.

**Figure 2:**
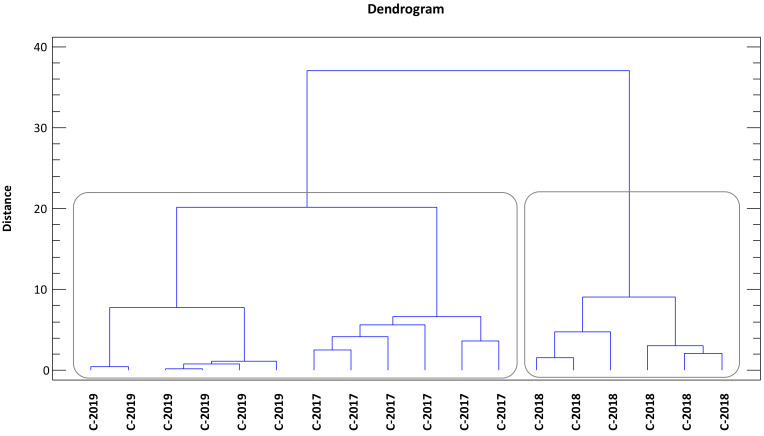
Dendogram grouping ‘Mansa de Figueiredo’ EVOOs according to the Euclidean distance by Ward’s method. C-2017: EVOOs from 2017 campaign; C-2018: EVOOs from 2018 campaign; C-2019: EVOOs from 2019 campaign.

Afterwards, another exploratory multivariate technique, HCA, was used to verify the robustness of the groups obtained by PCA. The cluster analysis was performed based on using Ward’s method and Euclidean as agglomerative and distance criteria, respectively. The dendrogram obtained (Figure 2) confirmed the previous group classification; that is, 12 phenolic compounds (variables) are capable of discriminating the studied EVOOs according to each crop season. Besides, additional information can be extracted from the dendrogram: choosing a relatively large and safe cutting value of 22 at the linkage distance in the dendrogram, all EVOOs from the 2019 crop season could be separated from the rest, which, in turn, could be further divided at the linkage distance of 10 into 2 subgroups, each one containing the samples from the 2017 and 2019 crop seasons. After having a look at this figure, it seems that the degree of similarity is higher between EVOOs from these two crop seasons. In this case, the role of RI in the olives picked up in the 2018 crop season is probably determinant in a such result.

**Figure 3:**
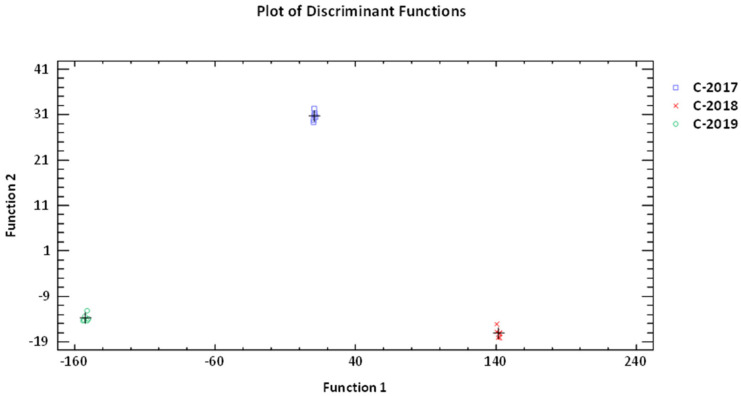
Plot showing discriminant functions of ‘Mansa de Figueiredo’ EVOOs according to crop year based on the concentration of 12 individual phenolic compounds (Function 1 vs. Function 2). C-2017: EVOOs from 2017 campaign; C-2018: EVOOs from 2018 campaign; C-2019: EVOOs from 2019 campaign.

Subsequently, the potential of employing a supervised multivariate method (s-LDA) to the dataset was tested without applying any variable reduction (12 predictor variables were entered). This classification method has been widely used in food research in order to obtain classification models [78]. With this methodology, the variable selection was performed by a leave-out cross-validation procedure to discard redundant information and to select only those variables that actually contributed to the increase in classification ability. The results showed that two statistically significant discriminant functions could be constructed at the 95.0% confidence level (explaining 96.83% and 3.17% of the data variability, respectively), and they achieved very satisfactory recognition and prediction abilities (Figure 3). Indeed, the model allowed the correct classification of 100% of the original grouped data. Thus, the phenolic compounds that mostly contributed to the differentiation in the EVOOs (mainly to the first discriminant function) in decreasing order of importance were: HTy-Ac > Ty > Dios > HTy > Pin.

Therefore, after collecting all this relevant information, it is possible to affirm that 12 representative phenolic compounds could be used as chemical markers to discriminate ‘Mansa de Figueiredo’ EVOOs according to crop year.

### 3.5. Effect of Crop Year on Olive Oil Antidiabetic Potential

The inhibition of the α-glucosidase enzyme is one of the contemporary therapeutic approaches in DM treatment. The inhibitors of α-glucosidase possess the ability to notably reduce or delay the concentrations of postprandial blood glucose [10,11].

Previous studies have reported that phenol-rich extracts from olive oil possess remarkable inhibitory activities on α-glucosidase [12,15,19,20].

However, to the best of our knowledge, no inhibitory effects on α-glucosidase activity for any olive cultivar, considering a period of three successive harvest years, have been investigated. Indeed, we believe it is relevant to evaluate the respective antidiabetic potential of olive oils through three consecutive seasons to ensure that it remains on the same scale over time, regardless of both climatic conditions and RI of olive fruit at harvesting time.

**Figure 4:**
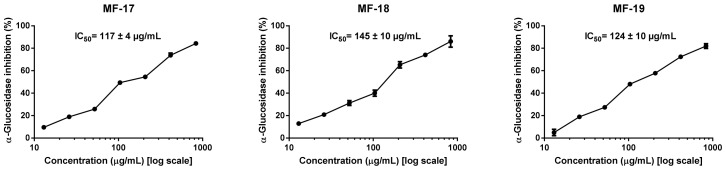
α-Glucosidase inhibition of phenol-rich extracts from ‘Mansa de Figueiredo’ EVOOs (MF-17, MF-18, and MF-19) obtained at three successive harvests. Results show the mean ± SD of 3 experiments of inhibition × 3 different phenolic extracts × 3 olive oils (*n* = 27 per crop year).

As shown in Figure 4, the present study provides evidence for the concentration-dependent inhibitory effect of the tested ‘Mansa de Figueiredo’ EVOOs on the α-glucosidase enzyme. Values of IC_50_ were calculated and displayed in the same figure as a measure of the inhibitory potency of the tested extracts.

The outcomes of the performed analysis revealed statistically significant differences among MF-18 EVOO and the other olive oils for the IC_50_ parameters examined. It was observed that phenol-rich extracts from ‘Mansa de Figueiredo’ EVOOs were slightly more active in the 2017 and 2019 crop years (IC_50_ = 117 and 124 μg of dry extract/mL, respectively), compared to the 2018 crop year (IC_50_ = 145 μg of dry extract/mL).

Moreover, the standard drug, namely acarbose (IC_50_ = 340 μg/mL), was used for comparison purposes with the tested olive oils. It is very interesting to remark that phenol-rich extracts from EVOOs were stronger inhibitors of α-glucosidase than acarbose, suggesting a promising inhibitory activity on this enzyme, regardless of the crop year.

The IC_50_ values evaluated herein (ranging from 117 to 145 μg of dry extract/mL) were considerably better than those described in the literature for EVOOs obtained from other varieties: ‘Cornicabra’ and ‘Picual’ varieties (246 and 291 μg of dry extract/mL, respectively) [19], and several Italian varieties (IC_50_ = 184–776 μg of dry extract/mL) [15]. This might be attributed to the fact that these olive oils were extracted using large-scale production. As previously mentioned, the use of different extraction procedures could affect the chemical composition of the extracts and, consequently, their biological activity. Recently, Reboredo-Rodríguez et al. [20] reported IC_50_ values ranging from 143 to 162 μg of dry extract/mL for EVOOs elaborated with the ‘Brava Gallega’ variety using the Abencor system. These authors also demonstrated that the RI might not be very decisive on the overall antidiabetic potential of these EVOOs. Thus, the IC_50_ value variability observed for the tested olive oils among three successive crop years could only depend on the pedo-climatic conditions of each evaluated crop season, which, in turn, affects their phenolic composition, as mentioned above [26,55].

It is well known that some phenolic compounds from olive oil, such as secoiridoids [15], oleuropein and HTyr [14,79], and flavonoids [18,80], are able to inhibit digestive enzymes, such as α-glucosidase [17].

Although the total phenolic content in olive oils was demonstrated to be statistically different for the analysed crop years, no correlation (based on a Pearson correlation test) between the inhibitory activity and phenolic content was observed. This fact could be explained because some phenolic compounds, which are present at relatively low concentrations, can be more active on the enzyme inhibition, illustrating the high specificity of the phenolic compounds–enzyme interaction, regardless of their concentration. In this respect, a work carried out by Figueiredo-González et al. [12] reported the negative correlation between Lut and Api and α-glucosidase inhibition. Besides, it is necessary to highlight that the possible synergistic or antagonistic effects between all phenolic compounds can also condition the inhibitory activity on this enzyme [17,81].

## 4. Conclusions

This research compiles the first deep evaluation of the inter-annual variability in the phenolic content and α-glucosidase enzyme inhibition of the Galician ‘Mansa de Figueiredo’ EVOOs. It is well known that the year of production is one of the main factors influencing the phenolic profile due to not only the climatic fluctuations but also the yearly variation in terms of the ripeness. In this regard, although the phenolic composition of ‘Mansa de Figueiredo’ EVOOs varied significantly throughout the different crop seasons, the substantial α-glucosidase inhibitory activity was maintained, demonstrating the efficacy of these olive oils as natural α-glucosidase inhibitors.

In addition, after applying several chemometric tools (PCA, HCA, and s-LDA), 12 of the 24 detected phenolic compounds could be used as chemical markers to discriminate ‘Mansa de Figueiredo’ EVOOs according to crop year.

Keeping all this in mind, this work can increase the interest in olive oil producers in this cultivar to obtain differentiated olive oils, contributing to their exploitation and valorisation as a way to preserve the olive heritage of Galicia.

## Figures and Tables

**Table 1:** Quality-related indices, sensory attributes, and composition of the studied olive oils.

	MF-17 (RI 3.5)	MF-18 (RI 5.0)	MF-19 (RI 2.3)	Regulated Values for EVOO (EU Reg 2568)
**Quality-related indices**				
**Free acidity (% oleic acid)**	0.18 ± 0.04 ^a^	0.15 ± 0.00 ^a^	0.18 ± 0.01 ^a^	≤0.80
**K_232_**	1.91 ± 0.02 ^b^	1.74 ± 0.03 ^a^	1.81 ± 0.02 ^a^	≤2.50
**K_270_**	0.19 ± 0.01 ^a^	0.16 ± 0.02 ^a^	0.16 ± 0.01 ^a^	≤0.22
**AK**	<0.01 ^a^	<0.01 ^a^	<0.01 ^a^	≤0.01
**Peroxide value (meq O_2_/kg oil)**	3.2 ± 0.3 ^a^	3.2 ± 0.1 ^a^	3.3 ± 0.3 ^a^	≤20.0
**Rancimat (h)**	43.3 ± 3.6 ^a^	44.1 ± 1.0 ^a^	40.7 ± 5.0 ^a^	
**Sensory analysis**				
***Positive attributes***				
Fruity	4.2	4.6	4.0	> 0.0
Bitter	4.0	3.4	3.7	
Pungent	4.4	4.1	4.5	
**Genuineness** **-related indices**				
***Fatty acid composition (% m/m methyl esters)***				
Myristic (C14:0)	0.012 ± 0.002 ^a^	0.008 ± 0.000 ^a^	0.010 ± 0.000 ^a^	≤0.03
Palmitic (C16:0)	10.36 ± 0.03 ^b^	10.44 ± 0.02 ^b^	9.81 ± 0.12 ^a^	7.50–20.00
Palmitoleic (C16:1)	0.620 ± 0.000 ^a^	0.650 ± 0.000 ^a^	0.493 ± 0.023 ^b^	0.30–3.50
Margaric (C17:0)	˂0.100 ± 0.007 ^a^	˂0.100 ± 0.000 ^a^	˂0.100 ± 0.000 ^a^	≤0.40
Margaroleic (C17:1)	˂0.100 ± 0.007 ^a^	˂0.100 ± 0.000 ^a^	˂0.100 ± 0.000 ^a^	≤0.60
Stearic (C18:0)	3.725 ± 0.021 ^c^	2.820 ± 0.014 ^b^	3.117 ± 0.120 ^a^	0.50–5.00
Oleic (C18:1)	74.75 ± 0.01 ^a^	76.57 ± 0.02 ^b^	78.72 ± 0.43 ^c^	55.00–83.00
**Linoleic (C18:2)**	8.900 ± 0.071 ^c^	7.935 ± 0.007 ^b^	6.040 ± 0.223 ^a^	2.50–21.00
Linolenic (C18:3)	0.565 ± 0.021 ^a^	0.580 ± 0.000 ^a^	0.640 ± 0.030 ^a^	≤1.00
Arachidic (C20:0)	0.480 ± 0.000 ^c^	0.380 ± 0.000 ^a^	0.470 ± 0.000 ^b^	≤0.60
Eicosenoic (C20:1)	0.305 ± 0.021 ^a^	0.315 ± 0.007 ^a^	0.337 ± 0.006 ^a^	≤0.50
Behenic (C22:0)	0.125 ± 0.007 ^b^	0.110 ± 0.000 ^a^	0.150 ± 0.000 ^c^	≤0.20
Lignoceric (C24:0)	˂0.100 ± 0.007 ^a^	˂0.100 ± 0.000 ^a^	˂0.100 ± 0.000 ^a^	≤0.20
*trans*-Oleic isomers C18:1 T	0.017 ± 0.001 ^a^	0.008 ± 0.000 ^a^	0.017 ± 0.006 ^a^	≤0.05
*trans*-Linoleic + *trans*-Linolenic	0.013 ± 0.001 ^a^	0.010 ± 0.000 ^a^	0.013 ± 0.006 ^a^	≤0.05
∑ SFA	14.796 ± 0.005 ^b^	13.853 ± 0.007 ^a^	13.560 ± 0.217 ^a^	
∑ MUFA	75.74 ± 0.04 ^a^	77.63 ± 0.01 ^b^	79.55 ± 0.41 ^c^	
∑ PUFA	9.465 ± 0.049 ^c^	8.515 ± 0.007 ^b^	6.680 ± 0.195 ^a^	
C18:1/C18:2	8.399 ± 0.068 ^a^	9.650 ± 0.011 ^a^	13.046 ± 0.546 ^b^	
∑ MUFA/∑ PUFA	8.002 ± 0.046 ^a^	9.117 ± 0.009 ^b^	11.916 ± 0.403 ^c^	
***Sterol relative amounts***				
Cholesterol (%)	0.245 ± 0.007 ^a^	0.120 ± 0.000 ^a^	0.167 ± 0.058 ^a^	≤0.5
Brassicasterol (%)	nd	nd	<0.100 ± 0.000	≤0.1
Campesterol (%)	2.240 ± 0.028 ^a^	2.250 ± 0.014 ^a^	2.433 ± 0.058 ^b^	≤4.0
Stigmasterol (%)	0.430 ± 0.014 ^a^	0.605 ± 0.007 ^b^	0.633 ± 0.058 ^b^	≤Campesterol
Apparent *β*-sitosterol (%)	94.75 ± 0.05 ^a^	95.33 ± 0.00 ^a^	95.13 ± 0.25 ^a^	≥93.0
Δ^7^-Stigmastenol (%)	0.340 ± 0.000 ^b^	0.170 ± 0.000 ^a^	0.333 ± 0.058 ^b^	≤0.5
Total sterols (mg/kg)	1131.0 ± 11.3 ^a^	1001.4 ± 14.3 ^a^	1645 ± 407.4 ^a^	≥1000
***Triterpenic alcohols***				
Erythrodiol + Uvaol (%)	2.430 ± 0.113 ^ab^	2.185 ± 0.021 ^a^	2.533 ± 0.058 ^b^	≤4.5
***Tocopherols (mg/kg)***				
*α*-tocopherol	184.05 ± 3.89 ^a^	273.00 ± 1.98 ^b^	303.30 ± 18.04 ^b^	
*β*-tocopherol	<0.1 ± 0.0	<0.1 ± 0.0	<0.1 ± 0.0	
*γ*-tocopherol	<0.1 ± 0.0	<0.1 ± 0.0	<0.1 ± 0.0	
*δ*-tocopherol	3.55 ± 0.64 ^a^	3.15 ± 0.07 ^a^	7.20 ± 0.10 ^b^	
Total tocopherols	187.60 ± 4.52 ^a^	276.20 ± 1.98 ^b^	313.50 ± 18.07 ^b^	

Values are expressed as mean ± standard deviation (*n* = 6, 3 olive oils × 2 determinations). In each row, different superscript letters mean significant statistical differences of the parameter under evaluation, at a 5% significance level (*p* < 0.05), according to multiple comparison Tukey’s HSD test.

**Table 2:** Total phenolic content and individual phenolic compounds (mg/kg) determined in ‘Mansa de Figueiredo’ EVOOs throughout different crop seasons.

**Total phenolic content by UV-VIS method**					**MF-17 (RI 3.5)**	**MF-18 (RI 5.0)**	**MF-19 (RI 2.3)**
**Folin-Ciocalteu**	mg GA/kg				675.4 ± 51.0 ^a^	705.9 ± 20.8 ^a^	793.1 ± 46.5 ^b^
***orto*** **-Diphenols**	mg GA/kg				196.7 ± 34.8 ^a^	226.5 ± 14.0 ^a^	214.5 ± 14.9 ^a^
**Antioxidant capacity**	µmol Trolox/kg				2678.4 ± 269.1 ^b^	1962.9 ± 73.7 ^a^	1982.3 ± 144.7 ^a^
**Phenolic profile by LC-MS method**		**Acronym**	**[M-H]^−^**	**Rt**	**MF-17 (RI 3.5)**	**MF-18 (RI 5.0)**	**MF-19 (RI 2.3)**
**Secoiridoids**	***Oleuropein derivatives***						
	Hydroxy oleacein (hydroxy decarboxymethyl oleuropein aglycone)	Hy-DOA	335	13.8	nd ^a^	16.2 ± 0.5 ^c^	2.2 ± 0.4 ^b^
	Oleacein (decarboxymethyl oleuropein aglycone)	DOA	319	14.7	350.5 ± 41.0 ^b^	221.3 ± 12.1 ^a^	542.1 ± 36.5 ^c^
	Oleuropein aglycone (isomer I)	OlAgl (Is I)	377	17.5	14.9 ± 2.1 ^b^	17.3 ± 1.2 ^c^	9.95 ± 0.8 ^a^
	Oleuropein aglycone (main peak)	OlAgl (main peak)	377	20.9	92.8 ± 7.8 ^c^	63.5 ± 5.6 ^b^	36.19 ± 2.8 ^a^
	Dehydro oleuropein aglycone	DH-OlAgly	375	20.9	nd ^a^	14.5 ± 0.1 ^c^	0.49 ± 0.04 ^b^
	Oleuropein aglycone (isomer II)	OlAgl (Is II)	377	21.7	13.0 ± 1.7 ^a^	21.7 ± 3.1 ^b^	12.2 ± 2.1 ^a^
	***Ligstroside derivatives***						
	Oleocanthal (decarboxymethyl ligstroside aglycone)	DLA	303	17.0	703.8 ± 59.1 ^b^	120.6 ± 9.7 ^a^	1060. 9 ± 123.0 ^c^
	Ligstroside aglycone (isomer I)	LigAgl (Is I)	361	20.8	98.1 ± 11.2 ^c^	11.4 ± 2.1 ^a^	21.3 ± 3.0 ^b^
	Ligstroside aglycone (main peak)	LigAgl (main peak)	361	23.3	226.8 ± 27.5 ^b^	162.2 ± 31.6 ^a^	204.7 ± 18.9 ^b^
	Ligstroside aglycone (isomer II)	LigAgl (Is II))	361	23.6	47.1 ± 9.7 ^a^	45.7 ± 8.7 ^a^	47.4 ± 5.5 ^a^
	**Sub-total**				1547.04	694.45	1937.60
**Simple phenols**	Oxidised Hydroxytyrosol	O-HTy	151	2.4	nd ^a^	14.00 ± 0.06 ^c^	0.029 ± 0.005 ^b^
	Hydroxytyrosol	HTy	153	6.5	17.8 ± 1.6 ^b^	19.5 ± 1.4 ^b^	3.0 ± 0.3 ^a^
	Tyrosol	Ty	137	8.2	10.6 ± 1.8 ^c^	5.7 ± 0.8 ^b^	2.5 ± 0.2 ^a^
	Hydroxytyrosol acetate	HTy-Ac	195	12.6	9.7 ± 0.4 ^b^	27.4 ± 2.3 ^c^	4.6 ± 0.3 ^a^
	**Sub-total**				**38.13**	**66.64**	**10.12**
**Flavonoids**	Luteolin	Lut	285	16.0	0.8 ± 0.2 ^a^	5.0 ± 0.4 ^c^	1.65 ± 0.08 ^b^
	Apigenin	Api	269	18.7	0.31 ± 0.05 ^a^	0.73 ± 0.06 ^b^	0.348 ± 0.004 ^a^
	Diosmetin	Dios	299	19.3	0.20 ± 0.03 ^b^	0.77 ± 0.04 ^c^	0.03 ± 0.02 ^a^
	**Sub-total**				**1.33**	**6.48**	**2.03**
**Phenolic acids**	Vanillic acid	Van	167	9.2	nd ^a^	0.09 ± 0.02 ^c^	0.07 ± 0.01 ^b^
	*p*-Coumaric acid	*p*-Cou	163	11.2	nd ^a^	0.33 ± 0.05 ^b^	0.058 ± 0.004 ^a^
	Ferulic acid	Fer	193	11.7	0.036 ± 0.001 ^c^	0.015 ± 0.002 ^b^	0.0099 ± 0.0001 ^a^
	**Sub-total**				**0.04**	**0.43**	**0.13**
**Lignans**	Pinoresinol	Pin	357	16.7	0.255 ± 0.048 ^b^	0.472 ± 0.044 ^c^	0.161 ± 0.006 ^a^
	**Sub-total**				**0.25**	**0.47**	**0.16**
**Non-phenolic but structurally related compounds**	Quinic acid	Quin	191	2.0	0.357 ± 0.024 ^b^	0.169 ± 0.019 ^a^	0.394 ± 0.037 ^b^
	***Elenolic acid derivatives***						
	Desoxy elenolic acid	Desoxy-EA	225	11.7	43.8 ± 4.6 ^a^	117.1 ± 5.2 ^b^	52.7 ± 5.0 ^a^
	Elenolic acid	EA	241	13.6	206.2 ± 19.4 ^b^	122.6 ± 24.6 ^a^	431.3 ± 36.6 ^c^
	**Sub-total**				**250.34**	**239.90**	**484.47**
	**TOTAL (mg each compound/kg)**				**1837.13**	**1008.39**	**2434.52**

Values are expressed as mean ± standard deviation (*n* = 6, 3 olive oils × 2 determinations). In each row, different superscript letters mean significant statistical differences of the parameter under evaluation, at a 5% significance level (*p* < 0.05), according to multiple comparison Tukey’s HSD test. nd: not detected.

## Data Availability

Data are contained within the article and Supplementary Materials.

## Supplementary Materials

The following supporting information can be downloaded at: https://www.mdpi.com/article/10.3390/antiox11071233/s1, Figure S1: Location (geographical coordinates and aerial view) of the three orchards where Mansa de Figueiredo cultivar is disseminated; Figure S2: Monthly rainfall (L/m^2^), minimum, maximum, and mean temperature (°C) and main features at the studied area during the three years covered in this study (2017–2019); Figure S3: Base peak chromatograms (BPCs) obtained using the optimum LC-ESI-IT MS conditions for ‘Mansa de Figueiredo’ EVOOs samples in the 2019 harvest year.
