# Supplementary material for: Singular Olive Oils from a Recently Discovered Spanish North-Western Cultivar: An Exhaustive 3-Year Study of Their Chemical Composition and In-Vitro Antidiabetic Potential
Source: Antioxidants (Basel). 2022 Jun 23;11(7):1233. doi: 10.3390/antiox11071233 (PMC9311737; doi:10.3390/antiox11071233)
Supplement: Supplementary file 1 [file antioxidants-11-01233-s001.zip › antioxidants-1774559-supplementary.pdf]

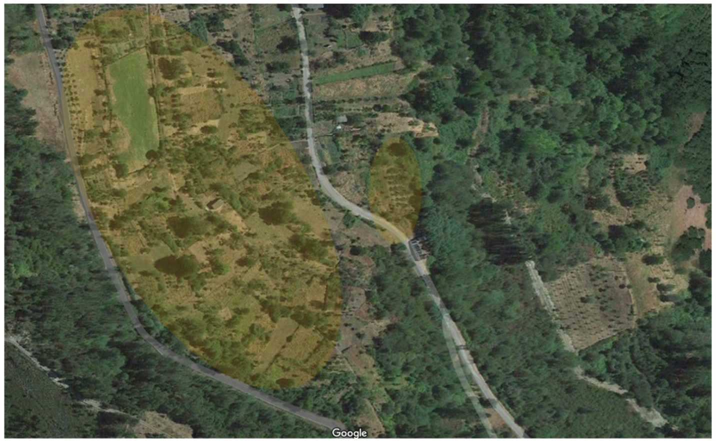

Orchard 1: N 42° 23' 20.2308", W 7° 12' 14.6736‘’
Orchard 2: N 42° 23' 17.502", W 7° 12' 18.162'"

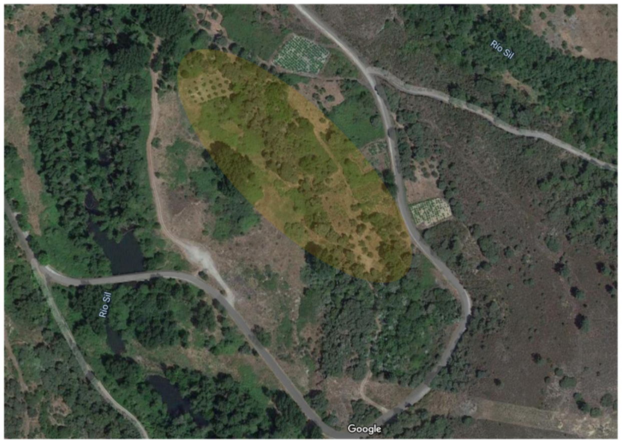

Orchard 3: N 42° 24' 25.6968", W 7° 13' 38.5824"

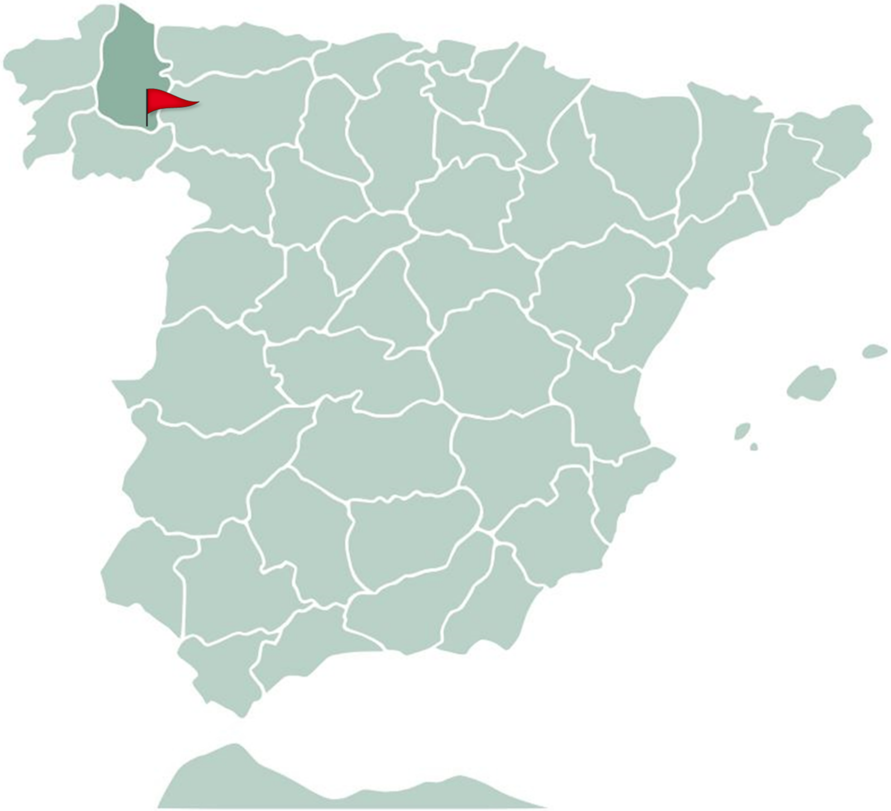

Figure S1. Location (geographical coordinates and aerial view) of the three orchards where Mansa de Figueiredo cultivar is disseminated.

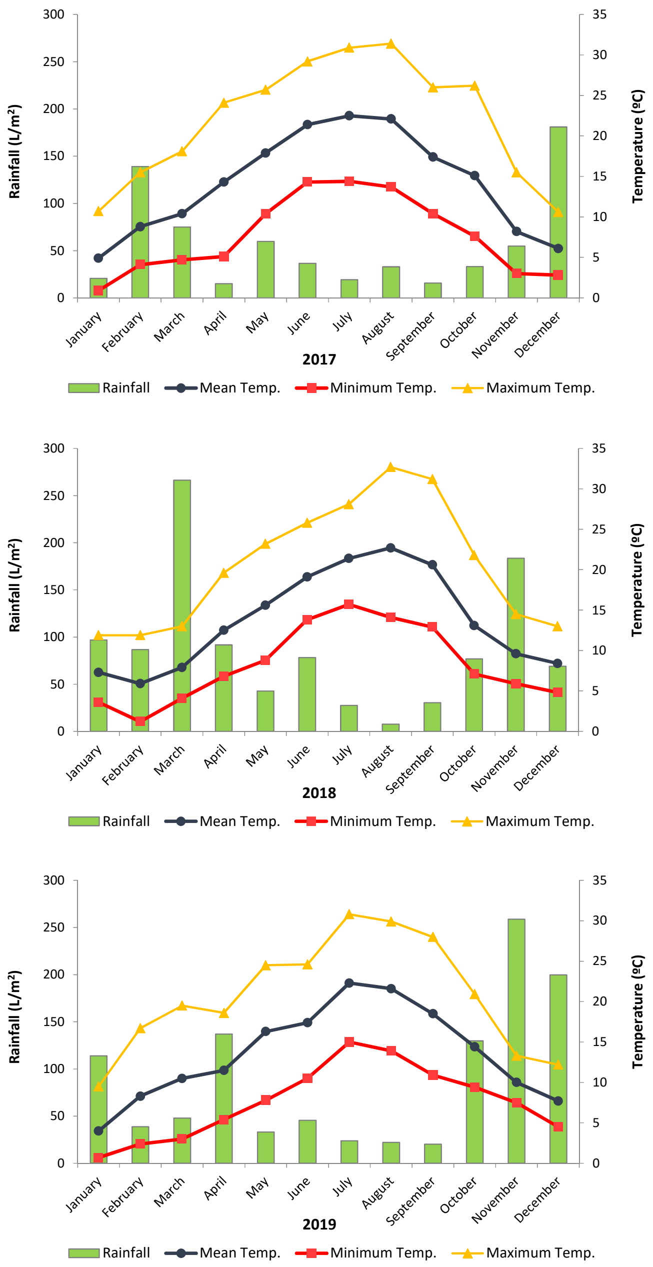

Season	2017	2018	2019
**December-February (winter)**	Dry and warm	Wet and not excessively cold	Dry and warm
**March-May (spring)**	Dry and very warm	Very wet and cold	Warm and normal rainfall
**June- August (summer)**	Dry and very warm	Wet and warm	Slightly cold and normal rainfall
**September-November (autumn)**	Very dry and warm	Very warm and normal rainfall	Very wet and warm autumn

Figure S2. Monthly rainfall (L/m²), minimum, maximum, and mean temperature (°C) and main features at the studied area during the three years covered in this study (2017-2019).

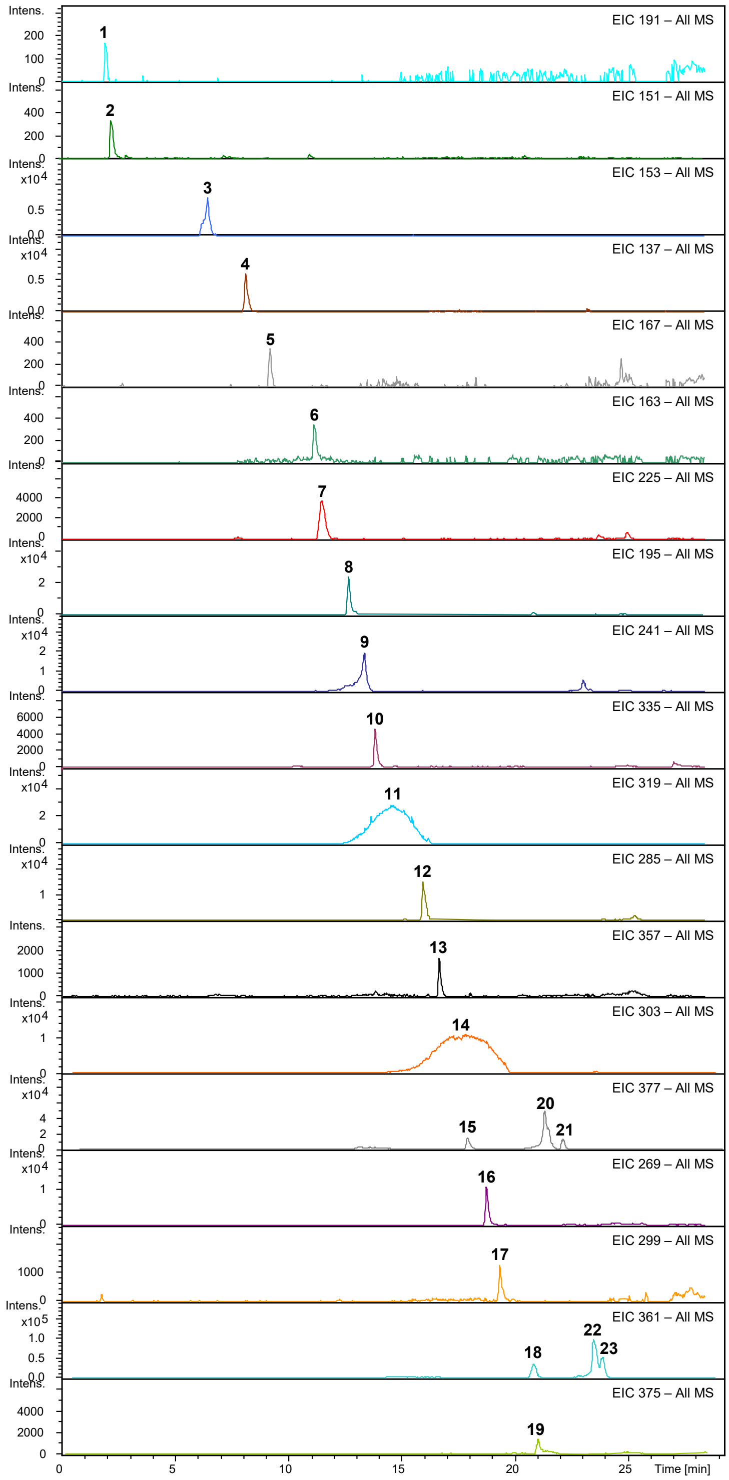

Figure S3. Base peak chromatograms (BPCs) obtained using the optimum LC-ESI-IT MS conditions for 'Mansa de Figueiredo' EVOOs samples in the 2019 harvest year. Peak numbers are as follows: **1**, quinic acid; **2**, oxidised hydroxytyrosol; **3**, hydroxytyrosol; **4**, tyrosol; **5**, vanillic acid; **6**, p-coumaric acid; **7**, desoxy elenolic acid; **8**, hydroxytyrosol acetate; **9**, elenolic acid; **10**, hydroxy oleacein, **11**, oleacein; **12**, luteolin; **13**, pinoresinol; **14**, oleocanthal; **15**, oleuropein aglycone (isomer I); **16**, apigenin; **17**, diosmetin; **18**, ligstroside aglycone (isomer I); **19**, dehydro oleuropein aglycone; **20**, oleuropein aglycone (main peak); **21**, oleuropein aglycone (isomer II); **22**, ligstroside aglycone (main peak); **23**, ligstroside aglycone (isomer II).
